# Accuracy of Intraocular Lens Power Calculation Formulas for Two Single-Piece Acrylic IOL Models in Eyes with a Shallow Anterior Chamber

**DOI:** 10.3390/bioengineering13101155

**Published:** 2026-10-02

**Authors:** Yalan Wang, Wei Lou, Lu Liu, Xinmin Lu, Qiang Wu, Da Long, Jianyan Hu

**Affiliations:** Department of Ophthalmology, Shanghai Sixth People’s Hospital Affiliated to Shanghai Jiao Tong University School of Medicine, Shanghai 200233, China

**Keywords:** intraocular lens power calculation, hydrophilic acrylic, intraocular lenses, hydrophobic acrylic, shallow anterior chamber

## Abstract

**Introduction:** This study evaluated whether the refractive prediction accuracy of conventional and new-generation intraocular lens (IOL) power calculation formulas differs between two single-piece acrylic IOL models in eyes with a shallow anterior chamber depth (ACD). **Methods:** In total, 100 eyes implanted with Softec HD IOLs (Lenstec) and 102 eyes implanted with SN60WF IOLs (Alcon) were included. All eyes had an ACD of ≤3.0 mm. The prediction error (PE) and mean absolute error (MAE) of 11 formulas, with and without optimized constants, were evaluated, including seven new-generation formulas (Barrett Universal II [BUII], Cooke K6 [K6], EVO 2.0, Hill-RBF 3.0, Hoffer QST, Kane, and Pearl-DGS) and four conventional formulas (Haigis, Hoffer Q, Holladay 1, and SRK/T). **Results:** The new-generation formulas (except K6) showed myopic PEs ranging from −0.108 to −0.181 D in the Softec group; most of these formulas (except Pearl-DGS and Hoffer QST) showed hyperopic PEs ranging from 0.120 to 0.310 D in the SN60WF group. Both before and after optimization, Hill-RBF 3.0 and Pearl-DGS yielded the lowest MAEs in the Softec and SN60WF groups, respectively. Before optimization, most formulas had MAEs > 0.43 D in the Softec group and <0.38 D in the SN60WF group; after optimization, the MAEs were 0.408–0.479 D and 0.334–0.377 D, respectively. **Conclusions:** The new-generation formulas with ULIB constants showed myopic and hyperopic PEs in the Softec and SN60WF groups, respectively. The SN60WF IOL model demonstrated better refractive prediction accuracy than the Softec model. Formula selection according to IOL model may help improve refractive outcomes in eyes with shallow ACDs.

## 1. Introduction

Recent advances in biometry, intraocular lens (IOL) design, and surgical techniques have facilitated the key goal of achieving targeted refractive outcomes in modern cataract surgery. Since the introduction of classic third-generation formulas three decades ago, IOL power calculation formulas based on various principles have continued to emerge. However, even the latest formulas do not show universal performance across all types of eyes [1,2]. A shallow anterior chamber, defined as an anterior chamber depth (ACD) of ≤3.0 mm [3], often leads to earlier cataract surgery because of associated hyperopia, narrow angles, or elevated intraocular pressure. Affected patients tend to have higher expectations for visual outcomes, but achievement of targeted refraction is more challenging due to the reduced accuracy of IOL power calculation formulas [4]. This anatomical characteristic, frequently accompanied by a short axial length (AL) and increased lens thickness (LT), can substantially influence prediction of the effective lens position (ELP), a critical variable that determines the refractive power of the IOL calculated by almost all formulas [5].

IOL design is another factor that can influence IOL power calculation. Therefore, each IOL model is assigned its own constant before market release. Formula developers and the ophthalmology community also recommend optimizing these constants in clinical practice to minimize systematic offsets in refractive predictions. Single-piece monofocal IOLs are a popular choice among surgeons. At our center, Softec HD (LENSTEC, Inc., St. Petersburg, FL, USA) and SN60WF IQ (Alcon Laboratories, Inc., Fort Worth, TX, USA) are two commonly used monofocal IOLs. Both are posterior chamber biconvex IOLs with 0° haptic angulation; they differ in several structural characteristics, including optic diameter, overall length, edge configuration, and acrylic material composition. Previous studies have suggested that differences in IOL characteristics can influence capsular adhesion and postoperative IOL position [6,7,8], potentially affecting refractive predictability. Furthermore, although modern IOL power calculation formulas incorporate increasingly sophisticated biometric parameters, their performance may still vary according to the IOL model implanted. Here, we hypothesized that Softec produces different refractive outcomes from SN60WF, particularly in eyes with a shallow ACD, an anatomically challenging condition for IOL power calculation.

Evidence remains limited regarding whether the accuracy of IOL power calculation formulas varies according to the implanted IOL model in eyes with a shallow ACD. Therefore, this study was designed to compare the accuracy of 11 conventional and new-generation formulas in eyes with shallow ACDs that were implanted with either the Softec HD or SN60WF IOL. We aimed to identify IOL model-associated patterns in formula performance that may assist formula selection in clinical practice, rather than to determine the independent effects of IOL material or individual design characteristics. Given the higher prevalence of shallow anterior chambers in the Chinese population [9], resolution of this research gap is needed to improve refractive outcomes in this population.

## 2. Methods

Approval for this retrospective study was granted by the Institutional Review Board of Shanghai Sixth People’s Hospital, and the study adhered to the tenets of the Declaration of Helsinki.

### 2.1. Selection Criteria and Data Collection

Consecutive patients with cataracts who underwent phacoemulsification with IOL implantation at the Department of Ophthalmology, Shanghai Sixth People’s Hospital, China, between April 2023 and December 2024 were identified through medical record review. All surgeries were performed by the same experienced surgeon. Inclusion criteria were as follows: (1) ACD ≤ 3.00 mm and (2) uneventful surgery with in-the-bag implantation of either the Softec HD or SN60WF IQ. In accordance with the recommendations of Abulafia et al. [10], only one eye per patient was included in the study. When both eyes satisfied the inclusion criteria, only the first operated eye was selected.

Exclusion criteria were as follows: (1) history of ocular surgery; (2) corneal disease, glaucoma, uveitis, lens dislocation, or ocular trauma; (3) combined surgical procedures during phacoemulsification; (4) postoperative best-corrected visual acuity <20/40; (5) any intraoperative or postoperative complication; and (6) incomplete follow-up or biometric data.

Preoperative optical biometry was performed using the IOLMaster 700 (Carl Zeiss Meditec AG, Jena, Germany) to obtain the following parameters: axial length (AL), ACD, central corneal thickness, keratometry (K), lens thickness (LT), and white-to-white distance. Postoperative manifest refraction was evaluated 4 weeks after surgery.

### 2.2. IOL Information

Softec HD is a single-piece posterior chamber biconvex IOL with a hydrophilic acrylic aspheric optic, 360° square edge, 0° haptic angulation, optic diameter of 5.75 mm, and overall length of 12.0 mm.

SN60WF IQ is a single-piece posterior chamber biconvex IOL with a hydrophobic acrylic aspheric optic, double square edge, 0° haptic angulation, optic diameter of 6.0 mm, and overall length of 13.0 mm.

### 2.3. Formula Calculations

To evaluate the performance of each formula in the clinical setting, we used optimized constants from the User Group for Laser Interference Biometry (ULIB).

In total, 11 formulas were analyzed, including four conventional formulas (Haigis, Hoffer Q, Holladay 1, and SRK/T) and seven new-generation formulas (Barrett Universal II [BUII], Cooke K6 [K6], Emmetropia Verifying Optical 2.0 [EVO 2.0], Hill-Radial Basis Function 3.0 [Hill-RBF 3.0], Hoffer QST, Kane, and Pearl-DGS). Conventional formulas were calculated using an Excel spreadsheet (Microsoft Corp., Redmond, WA, USA). New-generation formulas were calculated using the ESCRS IOL online calculators (available at https://iolcalculator.escrs.org, accessed on 10 November 2025) with all required parameters.

For each formula, prediction error (PE) was defined as the difference between the recorded postoperative spherical equivalent (SE) and the formula-predicted SE based on the implanted IOL power. The standard deviation (SD) of the PEs, median absolute error (MedAE), mean absolute error (MAE), and percentages of cases within ±0.25 D, ±0.50 D, ±0.75 D, and ±1.00 D of the target refraction were also calculated [11]. In accordance with recent literature, MAE was designated as the primary outcome measure [12].

Although constant optimization is not clinically recommended for eyes with specific anatomical characteristics, as proposed by Hoffer et al. [13], we also sought to compare the formulas in a research setting while minimizing the influence of systematic errors. Thus, each formula was also assessed after optimization. Following the method of Hoffer et al. [13], constants for the four conventional formulas were individually adjusted in Excel to set the PE to zero. Constants for the seven new-generation formulas were optimized online (available at https://ioloptimization.com/calculator, accessed on 12 November 2025) using the method proposed by Buonsanti et al. [14].

### 2.4. Statistical Analysis

Statistical analyses were performed using SPSS version 26.0 (IBM Corp., Armonk, NY, USA). Participants were grouped by IOL model (Softec vs. SN60WF) and subdivided into two ACD intervals: ACD ≤ 2.5 mm and 2.5 < ACD ≤ 3.0 mm [4]. Data normality was assessed using the Kolmogorov–Smirnov test. Single-group PEs were compared with zero using a one-sample t-test or Wilcoxon signed-rank test. Categorical variables (sex and eye laterality) were compared using the chi-square test. For interformula comparisons within groups, the Friedman test with post hoc Dunn–Holm correction was used, as recommended by a recent study for sample sizes of <150 [12]. Between-group MAEs for each formula were compared using unpaired t-tests, as proposed by Hoffer et al. [13]. The threshold for statistical significance was set at *p* < 0.05.

Using an online Power and Sample Size calculator (available at https://cqsclinical.app.vumc.org/ps/, accessed on 3 November 2025), we determined that 73 eyes per group were required to detect a 0.10 D difference in absolute prediction error, assuming an SD of 0.30 D, 80% power, and α = 0.05.

## 3. Results

### 3.1. Baseline Characteristics

This study included 202 eyes of 202 patients. Patient demographics and biometric data are presented in Table 1. No statistically significant between-group differences were observed in demographic characteristics or ocular biometric parameters, except for white-to-white distance and implanted IOL power.

### 3.2. Formula Comparisons

Table 2 shows the accuracy of the various formulas without constant optimization. With ULIB constants, most formulas produced significant myopic errors in the Softec group (*p* < 0.05), except for K6 (PE = 0.176 D), whereas most formulas produced significant hyperopic errors in the SN60WF group (*p* < 0.05). In the Softec group, Hill-RBF 3.0 (0.423 D), EVO 2.0 (0.425 D), and BUII (0.430 D) had the lowest MAEs; in the SN60WF group, Pearl-DGS (0.334 D), Hoffer Q (0.354 D), and Hoffer QST (0.355 D) had the lowest MAEs. In the Softec group, K6 produced a significantly higher MAE than BUII (*p* = 0.016). In the SN60WF group, K6 yielded a significantly higher MAE compared with EVO 2.0, Hill-RBF 3.0, Hoffer QST, Kane, Pearl-DGS, Hoffer Q, Holladay 1, and SRK/T (all *p* < 0.001).

Overall, the SN60WF group showed lower MAEs and MedAEs relative to the Softec group for a given formula. Between-group differences in MAE were statistically significant for Pearl-DGS, Hoffer QST, SRK/T, Holladay 1, and Hoffer Q (all *p* < 0.05).

Table 3 presents the accuracy of the various formulas after constant optimization. Hill-RBF 3.0 (0.408 D), EVO 2.0 (0.419 D), and Kane (0.432 D) yielded the lowest MAEs in the Softec group, whereas Pearl-DGS (0.334 D), EVO 2.0 (0.338 D), and Kane (0.340 D) yielded the lowest MAEs in the SN60WF group. Each formula in the SN60WF group had a significantly lower MAE than its counterpart in the Softec group (all *p* < 0.05). However, no significant interformula differences were observed within either group (all *p* > 0.05).

**Figure 1:**
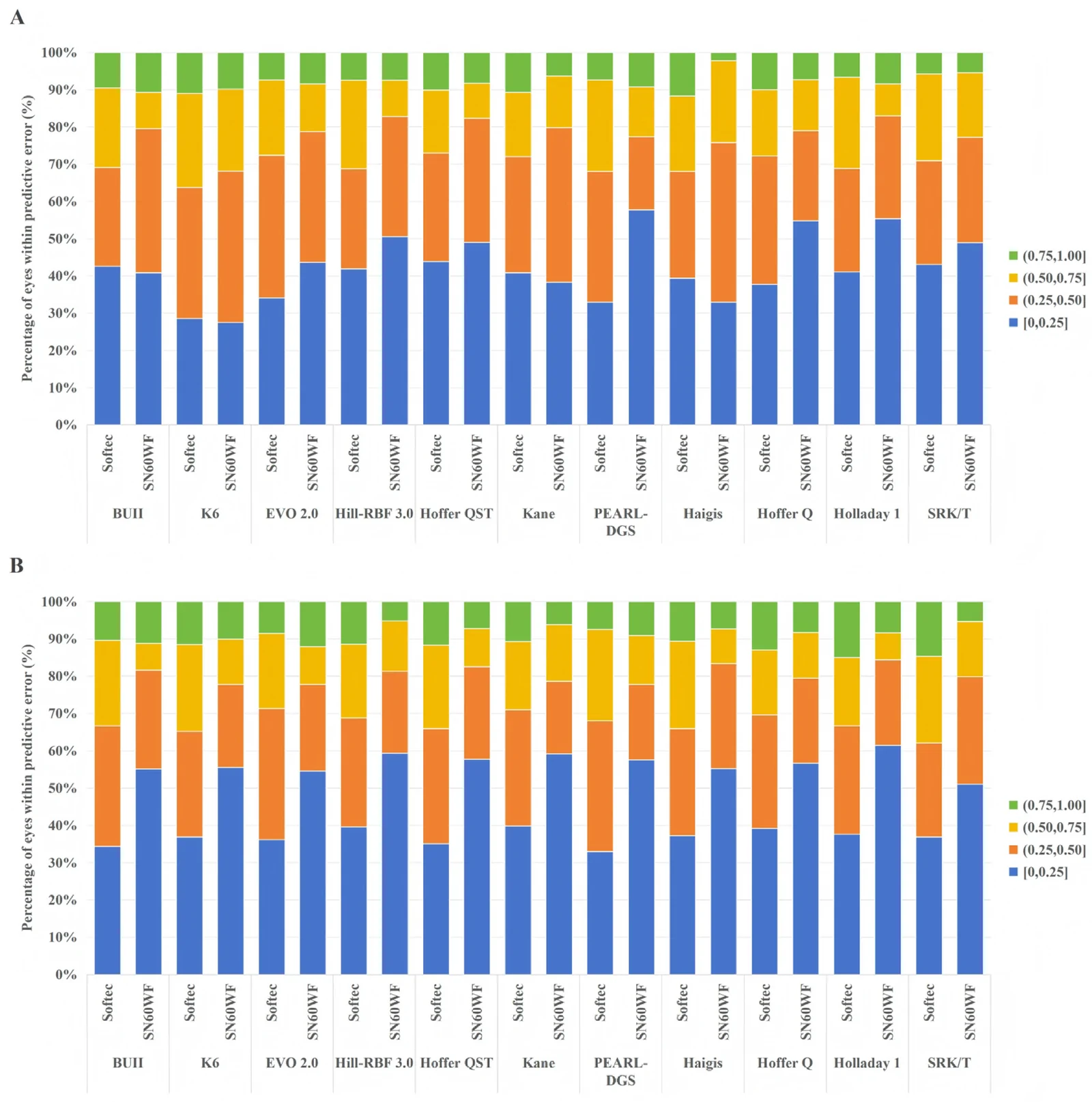
Stacked histograms showing the percentages of eyes with prediction errors within ±0.25 D, ±0.50 D, ±0.75 D, and ±1.00 D for the 11 formulas in the two intraocular lens groups: (**A**) before optimization and (**B**) after optimization.

Overall, the SN60WF group showed higher percentages of eyes with PEs within ±0.50 D across all formulas relative to the Softec group, regardless of constant optimization. Before optimization, with the exception of Haigis and K6, the percentages in the SN60WF group all exceeded 70% (range, 71–79%), whereas those in the Softec group ranged from 61% to 68%. After optimization, the percentage of eyes with PEs within ±0.50 D ranged from 74% to 79% in the SN60WF group, compared with 59% to 67% in the Softec group. Figure 1 displays cumulative proportions of eyes within ±0.25, ±0.50, ±0.75, and ±1.00 D of the predicted refraction.

### 3.3. ACD Subgroup Results

**Figure 2:**
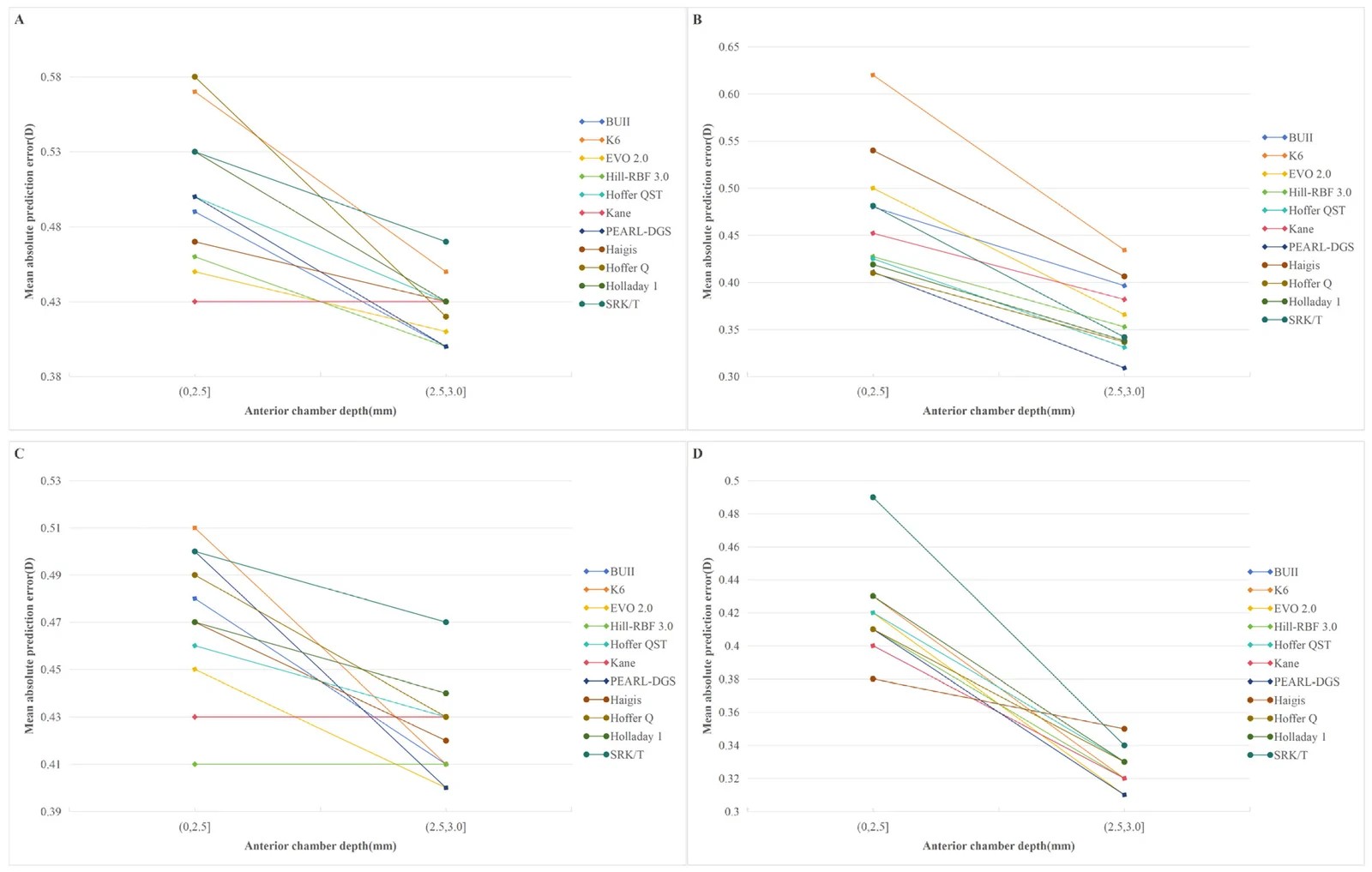
Mean absolute error in diopters for each formula, stratified by anterior chamber depth (ACD) interval (ACD ≤ 2.5 mm and 2.5 mm < ACD ≤ 3.0 mm). (**A**) Softec group and (**B**) SN60WF group before optimization; (**C**) Softec group and (**D**) SN60WF group after optimization. The numbers of eyes with ACD ≤ 2.5 mm were 35 in the Softec group and 25 in the SN60WF group. The numbers of eyes with ACD > 2.5 mm and ≤3.0 mm were 65 in the Softec group and 77 in the SN60WF group. In (**D**), the BUII and Kane lines overlap completely; the corresponding values for both formulas are reported in Table 4.

Table 4 and Figure 2 show the MAEs of the formulas in the two groups across the two ACD intervals. However, these findings should be considered exploratory because of the limited subgroup sample sizes.

Before optimization, in the Softec group, Kane and Pearl-DGS showed the numerically lowest MAEs for eyes with ACD ≤ 2.5 mm, whereas Hill-RBF 3.0 showed the lowest MAE for eyes with ACD > 2.5 to ≤3.0 mm (Figure 2A). In the SN60WF group, Pearl-DGS showed the lowest observed MAE in both ACD intervals (Figure 2B).

After optimization, in the Softec group, Hill-RBF 3.0 and Pearl-DGS yielded the numerically lowest MAEs for ACD ≤ 2.5 mm and ACD > 2.5 to ≤3.0 mm, respectively (Figure 2C). In the SN60WF group, Haigis and Pearl-DGS showed the lowest MAEs in the corresponding ACD intervals, respectively (Figure 2D).

## 4. Discussion

Eyes with a shallow anterior chamber often have a short AL and increased LT [15]. Congenital anatomical factors and age-related increases in LT play critical roles in the development of a shallow anterior chamber. In eyes with abnormal ocular anatomy, such as those with a short AL or relative anterior microphthalmos, a mismatch between AL and ACD can lead to inaccurate ELP prediction. In eyes with a shallow anterior chamber caused by lens-related factors, lens removal during cataract surgery may restore the postoperative ACD to a level close to that observed in normal eyes; however, this postoperative anatomical change might also compromise the performance of formulas that rely on preoperative ACD to predict ELP. Therefore, the accuracy of IOL power calculation in eyes with a shallow ACD has attracted increasing attention in recent years, particularly in Chinese individuals, because of population-specific anatomical characteristics [9]. These anatomical features may pose greater challenges for formula performance and IOL selection.

The present study demonstrated that the same IOL power calculation formulas exhibited different performance between the Softec HD and SN60WF groups in eyes with shallow ACDs. Although both Softec HD and SN60WF are single-piece posterior chamber biconvex acrylic IOLs with 0° haptic angulation, they differ in optic diameter, overall length, and edge configuration. Such design differences may influence the relationship between the IOL and capsular bag, affecting the predictability of postoperative ELP. Notably, both IOLs have an overall length of at least 12 mm, suggesting that a major effect of overall length on postoperative positioning is less likely [16]. Material-related biological responses, along with other IOL design characteristics, may contribute to the observed model-associated differences. However, the independent contribution of each factor cannot be determined from the present study. Compared with the hydrophobic acrylic material of SN60WF, the hydrophilic acrylic material of Softec HD has a greater capacity for water absorption and swelling due to the presence of HEMA-containing polymers [17,18,19]. These dimensional changes could theoretically affect the postoperative position of the optic and increase uncertainty in ELP prediction. Penetration of water molecules into the polymer network causes the material to swell to equilibrium, potentially altering the anteroposterior diameter of the optic of the implanted Softec IOL. Additionally, differences in material properties can influence capsular–IOL interactions. According to Liu et al. [6], at follow-up periods of ≥1 month, the incidence of posterior capsule adhesion was 100% for Softec, significantly higher than the 40.8% incidence for SN6ATX (which shares the same platform as SN60WF). Notably, Chang et al. [8] found that changes in postoperative ACD (defined as the distance from the corneal endothelium to the anterior surface of the IOL) were associated with IOL adhesion to the posterior capsule, noting that an IOL–capsular complex with tighter adhesion is more susceptible to axial movement under the influence of the zonular fibers. However, serial anterior-segment imaging was not performed in the present study to evaluate postoperative changes in IOL thickness or to directly assess the IOL–capsular bag interface. Therefore, water absorption-related swelling and capsular adhesion should be regarded only as potential mechanisms inferred from previous studies, rather than mechanisms demonstrated by our data. Moreover, because postoperative refraction was assessed at 4 weeks, the present findings should be interpreted as reflecting early refractive prediction accuracy, not long-term IOL stability. In this context, the observed difference in early refractive predictability between the two IOL models may reflect the combined effects of multiple IOL-related characteristics, rather than any single material- or design-related factor. Further studies comparing IOL models with controlled variations in individual design parameters are warranted to determine which characteristics most strongly influence formula performance.

The present study suggests that, for patients scheduled for Softec IOL implantation, surgeons may consider using newer-generation formulas (with the exception of K6 and Hoffer QST), regardless of whether optimized IOL constants are applied. Among the newer-generation formulas, Hill-RBF 3.0 and EVO 2.0 demonstrated relatively better performance for the Softec IOL model; both consistently exhibited the lowest MAEs regardless of correction for systematic error. However, when preoperative IOL power calculations are performed using Hill-RBF 3.0 and EVO 2.0, different considerations might be required. Given that EVO 2.0 showed a PE of only 0.044 D, the predicted refraction may be used directly for IOL power selection. In contrast, because Hill-RBF 3.0 exhibited a PE of approximately −0.17 D, surgeons should consider potential postoperative myopic shift during surgical planning to compensate for this systematic myopic bias. When IOL power calculations for SN60WF were performed using ULIB constants, the newer-generation formulas did not consistently outperform conventional formulas in terms of MAE. Specifically, when ranked by MAE from lowest to highest, the five most accurate formulas were Pearl-DGS, Hoffer Q, Hoffer QST, Holladay 1, and Hill-RBF 3.0, with corresponding MAEs of 0.334 D, 0.354 D, 0.355 D, 0.358 D, and 0.371 D, respectively. Although EVO 2.0, Kane, BUII, and K6 have demonstrated high accuracy in previous studies, these four newer-generation formulas did not show expected levels of performance in the present study. This discrepancy may be explained by the notable hyperopic PEs observed with all four formulas, ranging from 0.182 D to 0.310 D. After systematic errors were eliminated through constant optimization, newer-generation formulas (excluding Hoffer QST) demonstrated lower MAEs compared with conventional formulas, supporting the hypothesis that systematic error contributed to the inferior performance of newer-generation formulas when ULIB constants were used. Based on these findings, at centers using the IOLMaster 700 with ULIB constants, Hoffer Q may represent a reasonable option for IOL power calculation in patients with a shallow ACD undergoing SN60WF implantation, particularly when online calculators are not routinely used.

Unfortunately, this study did not identify a single formula that, when used with ULIB constants, consistently provided optimal refractive outcomes for both IOL models. This finding is particularly relevant for centers using the IOLMaster 700, which provides access to BUII and four conventional formulas, and in which surgeons may prepare two alternative IOL models for intraoperative selection based on surgical findings. Consequently, the achievement of optimal refractive outcomes may require surgeons or technicians to select formulas according to IOL model. To address this issue, we propose a potentially feasible approach: using the BUII formula with its integrated ULIB constants for both IOL models. For Softec, the MAE of BUII was surpassed only by those of Hill-RBF 3.0 and EVO 2.0, indicating that it outperformed conventional formulas (Table 2). For SN60WF, the predicted refraction could be adjusted by adding 0.21 D (the formula’s mean PE when using the ULIB constant). After correction of systematic error via constant optimization, the MAE of BUII was lower than those of the conventional formulas (Table 3). However, verification is needed concerning whether refractive outcomes obtained by adjusting for the mean PE are equivalent to those obtained using optimized constants.

This study has some limitations. First, the ACD-stratified analyses, particularly for eyes with ACD ≤ 2.5 mm, included a limited number of eyes and were not powered for definitive comparisons among formulas. These exploratory findings require confirmation in larger studies. Second, given ethnic variations in ocular biometry, our findings may not be directly generalizable to non-Chinese populations. Third, constant optimization is generally based on overall cataract populations, rather than specifically on eyes with shallow ACDs. Therefore, optimization tailored to this specific anatomical subgroup may reduce the external validity of the findings. Fourth, only two IOL models were evaluated; these models simultaneously differed in material composition and several design characteristics, including optic diameter and edge configuration. Therefore, the observed differences in formula performance cannot be attributed to any single IOL characteristic and may reflect the combined effects of material properties and structural design features. Further studies involving IOLs with controlled variation in individual characteristics are needed to clarify their independent contributions to refractive predictability.

## 5. Conclusions

The same formula may exhibit opposing systematic errors (myopic vs. hyperopic) across different IOL models in eyes with shallow ACDs; thus, surgeons should exercise caution when applying ULIB constants. To achieve more predictable refractive outcomes, surgeons may consider formula selection according to the IOL model selected for implantation. When IOL constants are optimized, most newer-generation formulas outperform conventional formulas. Overall, the SN60WF IOL model demonstrated better refractive predictability than the Softec HD IOL model in the present study, which may reflect the combined effects of multiple IOL-related characteristics.

## Figures and Tables

**Table 1:** Demographic and biometric data of all eyes.

Parameter	Softec (n = 100)	SN60WF (n = 102)	*p*
Age, years	73.42 ± 8.52 [50,90]	71.27 ± 11.04 [50,87]	0.383
Female gender, n (%)	77(77%)	75(73.5%)	0.568
Axial length, mm	23.15 ± 1.49 [20.98,33.01]	23.08 ± 1.02 [21.16,26.69]	0.830
Anterior chamber depth, mm	2.61 ± 0.33 [1.74,3.00]	2.67 ± 0.26 [1.82,3.00]	0.201
Mean keratometry, D	44.65 ± 1.60 [40.16,47.90]	44.41 ± 1.49 [41.42,47.57]	0.187
Corneal thickness, µm	537.35 ± 37.5 [451,663]	541.04 ± 30.9 [450,641]	0.122
Lens thickness, mm	4.72 ± 0.38 [3.55,5.55]	4.75 ± 0.40 [2.97,5.61]	0.588
White to white, mm	11.50 ± 0.40 [10.70,12.50]	11.63 ± 0.42 [10.70,13.38]	0.025 *
Implanted IOL power, D	21.21 ± 3.22 [2.00,26.50]	22.30 ± 2.27 [13,27]	0.007 *
Absolute postoperative refractive cylinder, D	0.91 ± 0.06 [0.00,2.50]	0.89 ± 0.06 [0.00,2.75]	0.880

D = diopter; IOL = intraocular lens. Values are presented as mean ± standard deviation; the range [minimum–maximum] is shown in brackets. * Significant difference between the two groups (*p* < 0.05).

**Table 2:** Accuracy of various intraocular lens power calculation formulas in the two groups before optimization.

Formulas	Softec HD (n = 100)							SN60WF (n = 102)							
	PE	SD	MAE	MedAE	Within ± 0.25 (%)	Within ± 0.50 (%)	Within ± 1.00 (%)	PE	SD	MAE	MedAE	Within ±0.25 (%)	Within ±0.50 (%)	Within ±1.00 (%)	*p* Value
BUII	−0.108	0.55	0.430	0.395	40	65	94	0.213 ^a^	0.51	0.417	0.335	38	74	93	0.725
K6	0.176 ^a^	0.58	0.494	0.352	26	58	91	0.310 ^a^	0.50	0.479 ^b^	0.404	25	62	91	0.730
EVO 2.0	0.044	0.54	0.425	0.360	32	68	94	0.182 ^a^	0.49	0.398 ^b^	0.351	41	74	94	0.405
Hill-RBF 3.0	−0.169 ^a^	0.52	0.423	0.330	39	64	93	0.120 ^a^	0.50	0.371 ^b^	0.270	47	77	93	0.163
Hoffer QST	−0.181 ^a^	0.56	0.453	0.358	39	65	89	−0.050	0.50	0.355 ^b^	0.264	47	79	96	0.032 ^*^
Kane	0.013	0.56	0.433	0.378	38	67	93	0.183 ^a^	0.49	0.398 ^b^	0.345	36	75	94	0.441
Pearl-DGS	−0.003	0.56	0.435	0.362	31	64	94	−0.002	0.48	0.334 ^b^	0.199	56	75	97	0.012 ^*^
Haigis	0.027	0.57	0.443	0.344	37	64	94	0.235 ^a^	0.53	0.440	0.369	30	68	91	0.687
Hoffer Q	−0.180 ^a^	0.58	0.474	0.385	34	65	90	0.034	0.51	0.354 ^b^	0.236	52	75	95	0.008 ^*^
Holladay 1	−0.188 ^a^	0.58	0.464	0.349	37	62	90	0.041	0.51	0.358 ^b^	0.233	52	78	94	0.046 ^*^
SRK/T	−0.240 ^a^	0.61	0.488	0.358	37	61	86	0.033	0.53	0.376 ^b^	0.285	45	71	92	0.059

BUII = Barrett Universal II; D = diopter; EVO = Emmetropia Verifying Optical; Hill-RBF = Hill-Radial Basis Function; MAE = mean absolute prediction error; MedAE = median absolute error; PE = prediction error; SD = standard deviation. * For a given formula, the MAE significantly differed between the two groups (*p* < 0.05). ^a^ Prediction error was significantly different from zero (*p* < 0.05). ^b^ Within-group comparisons were performed using the Friedman test with post hoc Dunn–Holm correction; Cooke K6 produced a significantly higher MAE than the other formulas (*p* < 0.0001).

**Table 3:** Accuracy of various intraocular lens power calculation formulas in the two groups after optimization.

Formulas	Softec HD (n = 100)							SN60WF (n = 102)							
	PE	SD	MAE	MedAE	Within ± 0.25 (%)	Within ± 0.50 (%)	Within ± 1.00 (%)	PE	SD	MAE	MedAE	Within ± 0.25 (%)	Within ± 0.50 (%)	Within ± 1.00 (%)	*p* Value
BUII	−0.0003	0.55	0.433	0.382	33	64	96	0.0003	0.50	0.341	0.217	53	78	96	0.018 *
K6	0.0000	0.57	0.443	0.366	35	62	95	0.0000	0.49	0.346	0.225	54	75	97	0.018 *
EVO 2.0	0.0006	0.54	0.419	0.335	34	67	94	0.0005	0.49	0.338	0.225	53	75	97	0.034 *
Hill-RBF 3.0	−0.0004	0.52	0.408	0.320	38	66	96	0.0007	0.50	0.343	0.220	56	76	94	0.048 *
Hoffer QST	0.0004	0.57	0.443	0.365	33	62	94	−0.0004	0.50	0.355	0.227	55	78	95	0.024 *
Kane	0.0009	0.56	0.432	0.355	37	66	93	0.0001	0.48	0.340	0.217	57	75	96	0.018 *
Pearl-DGS	0.0004	0.56	0.435	0.362	31	64	94	−0.0004	0.49	0.334	0.199	56	75	97	0.012 *
Haigis	−0.0003	0.57	0.442	0.353	35	62	94	−0.0004	0.52	0.355	0.234	52	78	94	0.013 *
Hoffer Q	0.0001	0.58	0.450	0.386	36	64	92	0.0007	0.50	0.351	0.235	54	75	95	0.011 *
Holladay 1	0.0001	0.59	0.452	0.380	35	62	93	0.0002	0.51	0.354	0.221	58	79	94	0.025 *
SRK/T	−0.0001	0.62	0.479	0.414	35	59	95	−0.0004	0.52	0.377	0.269	47	74	92	0.022 *

BUII = Barrett Universal II; D = diopter; EVO = Emmetropia Verifying Optical; Hill-RBF=Hill-Radial Basis Function; MAE = mean absolute prediction error; MedAE = median absolute error; PE = prediction error; SD = standard deviation. * For a given formula, the MAE significantly differed between the two groups (*p* < 0.05).

**Table 4:** Mean absolute errors of formulas across two anterior chamber depth intervals for two intraocular lens types.

IOL Types	Softec HD				SN60WF			
	Before Optimization		After Optimization		Before Optimization		After Optimization	
Formulas ACDintervals (mm)	(0, 2.5] ^a^	(2.5, 3.0] ^b^	(0, 2.5] ^a^	(2.5, 3.0] ^b^	(0, 2.5] ^a^	(2.5, 3.0] ^b^	(0, 2.5] ^a^	(2.5, 3.0] ^b^
BUII	0.49	0.40	0.48	0.41	0.48	0.40	0.40	0.32
K6	0.57	0.45	0.51	0.41	0.62	0.43	0.43	0.32
EVO 2.0	0.45	0.41	0.45	0.4	0.50	0.37	0.42	0.31
Hill-RBF 3.0	0.46	0.4	0.41	0.41	0.43	0.35	0.41	0.32
Hoffer QST	0.50	0.43	0.46	0.43	0.43	0.33	0.42	0.33
Kane	0.43	0.43	0.43	0.43	0.45	0.38	0.40	0.32
Pearl-DGS	0.5	0.4	0.5	0.4	0.41	0.31	0.41	0.31
Haigis	0.47	0.43	0.47	0.42	0.54	0.41	0.38	0.35
Hoffer Q	0.58	0.42	0.49	0.43	0.41	0.34	0.41	0.33
Holladay 1	0.53	0.43	0.47	0.44	0.42	0.34	0.43	0.33
SRK/T	0.53	0.47	0.50	0.47	0.48	0.34	0.49	0.34

Anterior chamber depth; BUII = Barrett Universal II; Hill-RBF=Hill-Radial Basis Function; D = diopter; EVO = Emmetropia Verifying Optical; IOL = intraocular lens. ^a^ The numbers of eyes with ACD ≤ 2.5 mm were 35 in the Softec group and 25 in the SN60WF group. ^b^ The numbers of eyes with ACD > 2.5 mm and ≤ 3.0 mm were 65 in the Softec group and 77 in the SN60WF group.

## Data Availability

The datasets generated and/or analyzed during the current study are not publicly available due to patient privacy and confidentiality considerations but are available from the corresponding author on reasonable request, subject to institutional and ethical restrictions.
